# Pre-attentive modulation of brain responses to tones in coloured-hearing synesthetes

**DOI:** 10.1186/1471-2202-13-151

**Published:** 2012-12-14

**Authors:** Lutz Jäncke, Lars Rogenmoser, Martin Meyer, Stefan Elmer

**Affiliations:** 1 Division Neuropsychology, Institute of Psychology, University of Zurich, Binzmühlestrasse 14/25, Zurich CH-8050, Switzerland; 2 Center for Integrative Human Physiology, Zurich, Switzerland; 3 International Normal Aging and Plasticity Imaging Center (INAPIC), Zurich, Switzerland; 4 Research Unit “Plasticity and learning in the aging brain”, University of Zurich, Zurich, Switzerland

**Keywords:** Coloured-hearing synesthesia, Crossmodal integration, EEG, Mismatch negativity, Auditory cortex

## Abstract

**Background:**

Coloured-hearing (CH) synesthesia is a perceptual phenomenon in which an acoustic stimulus (the inducer) initiates a concurrent colour perception (the concurrent). Individuals with CH synesthesia "see" colours when hearing tones, words, or music; this specific phenomenon suggesting a close relationship between auditory and visual representations. To date, it is still unknown whether the perception of colours is associated with a modulation of brain functions in the inducing brain area, namely in the auditory-related cortex and associated brain areas. In addition, there is an on-going debate as to whether attention to the inducer is necessarily required for eliciting a visual concurrent, or whether the latter can emerge in a pre-attentive fashion.

**Results:**

By using the EEG technique in the context of a pre-attentive mismatch negativity (MMN) paradigm, we show that the binding of tones and colours in CH synesthetes is associated with increased MMN amplitudes in response to deviant tones supposed to induce novel concurrent colour perceptions. Most notably, the increased MMN amplitudes we revealed in the CH synesthetes were associated with stronger intracerebral current densities originating from the auditory cortex, parietal cortex, and ventral visual areas.

**Conclusions:**

The automatic binding of tones and colours in CH synesthetes is accompanied by an early pre-attentive process recruiting the auditory cortex, inferior and superior parietal lobules, as well as ventral occipital areas.

## Background

Coloured-hearing synesthesia (CHS) is a perceptual phenomenon in which auditory stimuli cause additional colour experiences. To date, different forms of CHS have been reported, comprising tone-colour [1,2], spoken word-colour [1], music-colour [3], or general auditory-colour synesthesia [4]. The common denominator of all these synesthesia variants is a close relationship between auditory and visual perceptual representations. Furthermore, these synesthetic experiences (as all other synesthetic forms) are fast, non-suppressible, and mostly unidirectional [5], although cases of bidirectional synesthesia have been reported [6,7].

Two different models^a^ are mainly discussed to explain the neurophysiological underpinnings of all variants of synesthesia: the two-stage cross-activation / hyper-binding model [8], and the disinhibited feedback model [9,10]. The two-stage cross-activation / hyper-binding model was proposed on the basis of fMRI studies conducted with grapheme-colour synesthetes, and primarily relies on the physical closeness between the involved processing areas (e.g., grapheme and colour areas) [8]. According to this framework, the grapheme and colour processing areas (V4) are functionally and/or anatomically strongly interconnected. Consequently, this aberrant connectivity should result in co-activation of these areas during grapheme processing. Both perceptions are then bound together by parietal regions, resulting in hyper-binding. For CH synesthesia this would imply strong anatomical and/or functional connections between auditory and visual (as well as parietal) areas, and therefore rely on cross-activation across a quite long distance. This specific model has received some support from grapheme-colour as well as from CHS [11-18]. Otherwise, the disinhibited feedback model is based on studies demonstrating specific forms of acquired and congenital synesthesia rather than on brain imaging data (first mentioned by Armel and Ramachandran [9] and summarized by Grossenbacher & Lovelace [10]). The disinhibited feedback model suggests that synesthesia results from disinhibited feedback from higher-level cortical areas in the processing hierarchy. With respect to CHS, this would imply that higher-level cortical areas collect information transmitted from the auditory cortex, and project this information to the brain areas eliciting the concurrent perception (for CHS to the colour area V4). On the basis of the two models, different predictions can be drawn with respect to the timing of neural activations associated with the concurrent perception. The cross-activation / hyper-binding model proposes simultaneous activation of the brain areas involved in processing the inducer and the concurrent. By contrast, the disinhibition model proposes that the neural activation associated with the processing of the inducer should precede that associated with the processing of the concurrent [19]. However, none of these models makes any assumptions about the activation of the brain areas processing the inducer.

CHS (as all other forms of synesthesia) can be seen as a special variant of audiovisual (AV) integration. In fact, even though visual stimuli are not physically present, they are perceived during auditory stimulation. Recent research in non-synesthetes has been dedicated to identify the time point during perception at which cross-modal stimuli are merged to a single percept. However, it is currently debated whether cross-modal integration happens at very early or rather later processing stages. One idea is that AV stimuli would impact primary sensory areas due to feedback loops from higher-order multisensory areas [20]. Other researchers rather proposed that AV stimuli would influence the early stages of sensory processing by feedforward inputs and lateral connections between the involved primary sensory areas [21]. Early and automatic AV integration mechanisms have been demonstrated in non-synesthetes in the context of the McGurk illusion effect [22-24] or even in skilled readers while processing graphemes [25]. Of particular interest for the present work is that these previous studies investigated AV interactions by evaluating a pre-attentive component of the event-related potential (ERP), namely the mismatch negativity (MMN). The MMN indicates an automatic and pre-attentive auditory deviance, and one of its major sources is located in the auditory cortex (primary and secondary). However, also sources extending into brain areas outside the auditory cortex (superior temporal sulcus, temporal and parietal areas) have been reported [26-28]. The MMN is evoked between 100 and 250 ms after stimulus onset when a rarely presented sound deviates (the deviant) from a frequently presented standard sound (the standard) in one or more dimensions. It is important to mention that the MMN is even evoked when the subjects focus attention on other aspects than the auditory stimuli [29,30]. Therefore, it is assumed that the MMN reflects pre-attentive processing. Previous studies which made use of the MMN for examining AV interactions have repeatedly shown that the amplitude of the MMN is larger in response to auditory stimuli which are automatically integrated with visual features. For example, skilled readers with several years of practise demonstrate enhanced MMN amplitudes in response to phonemes when they are presented simultaneously with the corresponding graphemes [25].

Since none of the previous synesthesia studies made any assumptions about the activation of the brain areas involved in processing the inducer, in the present work we used the MMN in order to examine whether tone-colour associations modulate brain activity in the auditory-related cortex of CH synesthetes. In other words, we were interested in examining whether synesthetic AV processing happens pre-attentively at very early stages of auditory processing. This is of particular interest for the question whether synesthetic experiences are driven by early (perceptual, bottom-up) or late (cognitive, top-down) processing steps. In this context, recent work has proposed that both bottom-up and top-down processes might contribute to synesthesia [31]. For example, Ramachandran and Hubbard [32] classified grapheme-colour synesthetes as “higher” synesthetes for whom the grapheme “concept” is critical, and “lower” synesthetes for whom the “percept” of the physical grapheme is necessary to elicit synes-thetic experience. According to this classification, “lower” synesthetes would show greater neurophysiological modulation in early processing stages, whereas “higher” synesthetes would show greater neurophysiological modulation in later ones.

Based on previous work showing that AV double deviations (i.e., the simultaneously presented visual and auditory stimuli deviate from the simultaneously presented visual and auditory standards) result in larger MMN [25], we expect to find larger MMN responses to tones in CH synesthetes compared to non-synesthetes. In fact, for CH synesthetes each acoustic deviant is automatically processed as double deviation. This means that tones deviating from the standard will automatically induce the perception of a different colour (double deviation: tone and concurrent colour of the deviant diverge from the standard). Hence, the larger the double deviation is, the larger the MMN should be. However, small frequency differences between the standard and the deviant often induce the same colour experience in CH synesthetes. In this specific case, the MMN should not differ between CH synesthetes and non-synesthetes since the deviant only differs in the auditory dimension while the colour perception does not change.

## Results

### Age, general cognitive capability, and musical aptitudes

CH synesthetes and control subjects did not differ in age nor in general cognitive capability (age *t*_20_ = 0.326, p = .74; cognitive capability *t*_20_ = 1.391, p = .18; *t*-tests for independent samples, two-tailed). Furthermore, we did not reveal group differences in the tonal (*t*_20_ = 1.173, p = .25) nor in the rhythmical (*t*_20_ = 0.568, p = .57) parts of the test for musical aptitudes (*t*-tests for independent samples, two tailed).

### Absolute pitch (AP) test

For the two subjects per group who claimed to have AP, verification was conducted by using an in-house test [33]. The accuracy was evaluated by counting the total number of correct responses. Semitone errors were taken as incorrect responses in order to increase the discriminatory power of the test (percentage of correct responses; S 6 = 62.9%, S 11 = 64.8%; C 1 = 99%, C 2 = 74%). The two control subjects apparently performed better than the two synesthetes on the AP test.

### Test of genuineness of synesthesia

**Figure 1:**
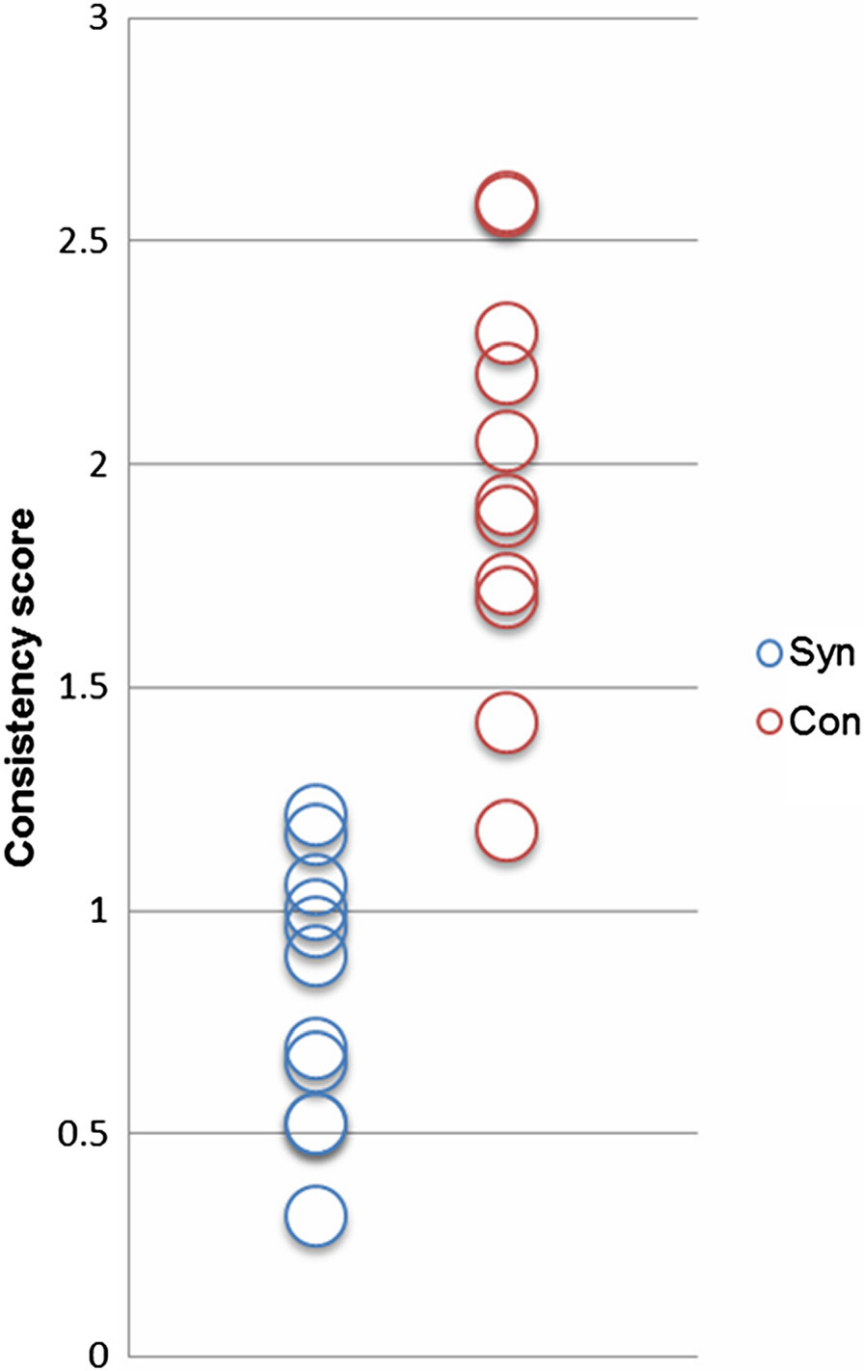
**Colour consistency scores of each subject and both groups. **During the colour consistency test, participants were presented with randomly presented tones and had to choose from a colour palette the colours that best matched their synesthetic experience. Blue = CH synesthetes; red = controls.

In order to verify that the subjects were indeed CH synesthetes, we performed a tone-colour consistency test in which the subjects were required to select colours from a colour palette according to their associations with 13 piano tones (range 261–523 Hz, presented three times in a randomized order) [34]. According to this test, all synesthetes reported vivid, immediate, and distinct colour perceptions which were significantly more consistent than associations reported by control subjects in response to the tones (*t*_20_ = −7.064, p < 0.001; *t*-test for independent samples, two-tailed). Figure 1 shows the consistency scores of each subject and the two groups.

### Synesthetic colours induced by tones

The concurrent colours induced by the different tones are depicted in Additional file 1: Table S1, separately for the standard (tone A) and the four deviants (differing from the standard in 1/10, 1/4, 1, and 9 semitone steps). As visible in Additional file 1: Table S1, with the exception of subjects S1 and S2 as well as partly S3, all synesthetes perceived similar concurrent colours in response to the 1/10-semitone deviant and the standard. For the deviants differing in 1/4 or 1 semitone from the standard, the synesthetes experienced different colours than during the presentation of the standard. Interestingly, five of the synesthetes perceived identical colours for the 1/4 or 1 semitone deviants (S2, S3, S4, S5, and S7). In addition, the concurrent colour perception of S6 was nearly similar (a darker blue) for the 1/4-semitone deviant and for the 1-semitone deviant. Table 1 depicts the mode of experiencing synesthetic colors of each subject.

**Table 1:** Individual modes of synesthetic colour experience

	**Spatial locations**			**Interferences**		
	**External**	**Internal**	**Gaze centred**	**Posture**	**Sound source**	**Unaffected**
S1		x				x
S2		x				x
S3		x				x
S4					x	
S5	x				x	
S6		x				x
S7		x		x	x	
S8	x				x	
S9		x		x	x	
S10		x	x		x	
S11		x				x

Most subjects described the tone-induced synesthetic colours occurring internally, while a few experienced them externally. Apart from this polarity, one subject described the synesthetic colours occurring in a gaze-centred manner. Furthermore, for many subjects, the spatial locations varied depending on their posture and/or the sound source. Many subjects also reported their synesthetic colours being unaffected by interfering aspects.

### Electrophysiological results

**Figure 2:**
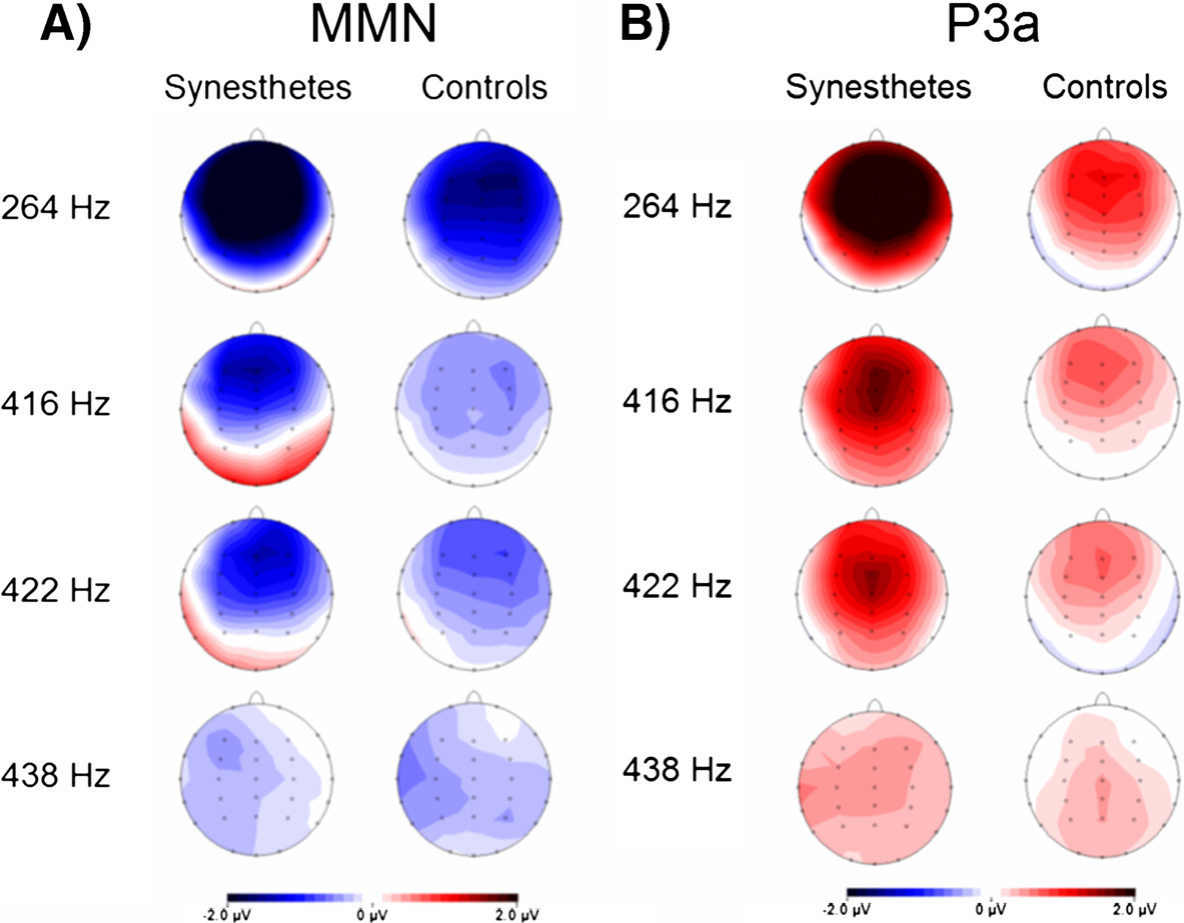
Scalp distribution of averaged MMN (A) and P3a (B) amplitudes for both groups and all four deviants.

In Figure 2A, the topographies of the MMNs are presented separately for the two groups (synesthetes and non-synesthetes) and all deviant conditions. In both groups the most prominent negative deflections reflecting the MMN were elicited at fronto-central rather than at posterior or occipital scalp sites. Figure 2B shows the positive fronto-central scalp distribution associated with the P3a (novelty P3) component.

**Figure 3:**
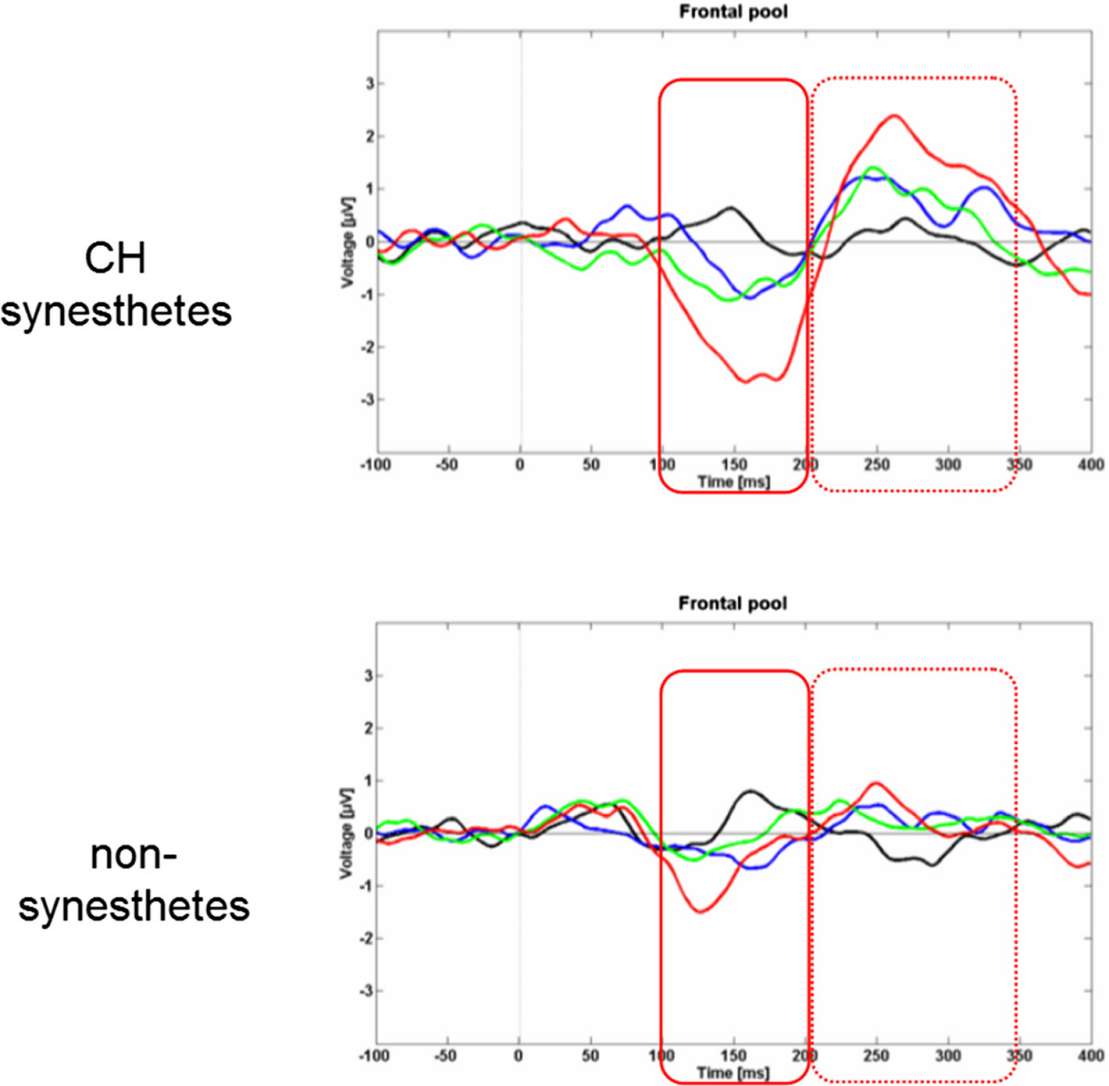
**Grand average of the MMN and P3a at the frontal pool of electrodes separately for CH synesthetes (upper part) and non-synesthetes (lower part). **The MMN and P3a are shown for the four deviants (black: 438, blue: 422, green: 416, and red: 264 Hz tones). The red rectangle in solid line indicates the time frame for the MMN while the red rectangle with the dotted line indicates the time frame for the P3a.

**Figure 4:**
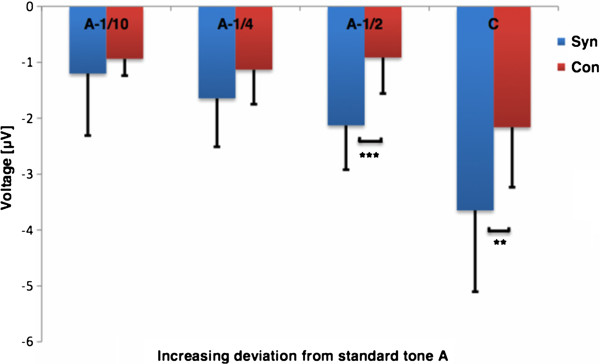
**Mean MMN amplitudes for both groups and for all deviants. **Significant group differences are indicated by asterisks (*** p < .001, ** p < .01).

The MMN (red rectangle with solid line) and P3a waveforms (red rectangle in dotted line) are shown in Figure 3, separately for synesthetes (upper part) and non-synesthetes (lower part) and the four deviant conditions. The mean (peak) MMN amplitudes from the frontal ROI are shown in Figure 4. In a first analysis, we tested whether the MMN amplitudes significantly differed from baseline by using one-sample *t*-tests (one-tailed). This procedure confirmed genuine MMNs for all deviant conditions and the two groups. The MMN amplitudes were further evaluated by computing a 2 × 4 ANOVA (2 *groups*, 4 *deviants*, with repeated measurements for the last factor). This statistical procedure led to significant *group* (F_1,20_ = 11.044, p < .01), *deviant* (F_3,20_ = 39.891, p < .001), and *group x deviant* (F_1,20_ = 5.984, p < .05) effects. To further explore the *group × deviant* interaction effect, we compared the MMN amplitudes of the two groups across the four deviant conditions by performing *t*-tests for independent samples (one-tailed, Bonferroni-Holm corrected). These post-hoc *t*-tests revealed significant between-group differences only in response to the piano tones of 416 Hz (1 semitone, t_20_ = 3.939, p < 0.001) and 264 Hz (9 semitone, *t*_20_ = 2.726, p < 0.01). Furthermore, in order to preclude that the two AP possessors per group may have influenced the data, we additionally performed a 2 × 4 ANOVA by excluding the subjects with AP. This supplementary statistical analysis clearly evidenced that the AP possessors did not influence the data (*group* (F_1,16_ = 14.232, p < .001) ; *deviant* (F_3,16_ = 32.65, p < .001); *group x deviant* (F_1,16_ = 7.062, p < .05). In line with our hypothesis, the CH synesthetes showed principally larger MMN amplitudes than the control subjects in response to the piano tones belonging to a novel tone category than the standard tone, and therefore more likely inducing a change in colour experience. MMN latencies were also subjected to a 2 × 4 ANOVA for repeated measurements on the second factor (two *groups* as the grouping factor and the 4 *deviants* as repeated measurement factor). This ANOVA revealed a significant main effect for *group* (F_1,20_ = 5.3, p < .05) and *deviant* (F_3,20_ = 7, p < 0.05). There was no interaction between *group* and *deviant* (F_1,20_ = 2, p = .23). The main effect of *group* was qualified by on average larger MMN latencies for the synesthetes compared to the non-synesthetes. The main effect for *deviant* originated from increased MMN latencies with increasing deviant magnitudes.

**Figure 5:**
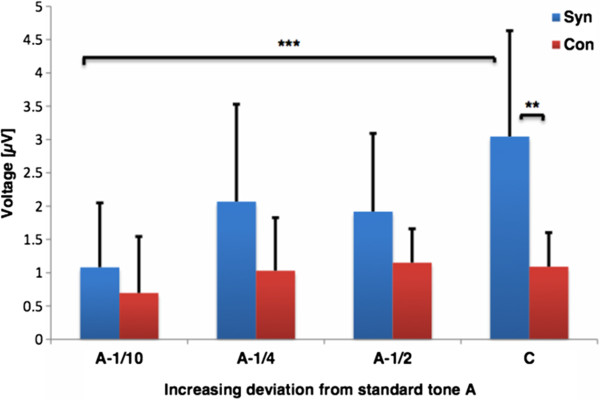
**Mean P3a amplitudes for both groups and all deviants. **Significant group differences are indicated by asterisks (*** p < .001, ** p < 0.01).

In a similar way as done for the MMN, we evaluated whether P3a amplitudes significantly differed from zero (one-sample *t*-test). Results clearly indicated the presence of genuine P3a responses in both groups and the four deviant conditions. The peak amplitudes of the P3a were subjected to a 2 × 4 ANOVA with the between-subjects factor *group* (synesthetes vs. non-synesthetes) and the repeated measurement factor *deviant*. This ANOVA revealed main effects for *group* (F_1,20_ = 15.9, p < 0.01) and *deviant* (F_3,20_ = 20.5, p < 0.001) as well as a significant interaction between *group* and *deviant* (F_1,20_ = 8.1, p < 0.05). The *group* x *deviant* interaction originated from larger P3a amplitudes in the synesthetes in comparison to non-synesthetes in response to the deviant with the largest frequency difference (264 Hz, t_20_ = 3.9, p < .01). Furthermore, from Figure 5 it becomes visible that in the synesthetes the P3a amplitudes increased as a function of the deviant magnitudes (*t*_10_ = 4.5, p < .001). For the non-synesthetes the P3a amplitudes were comparable across all deviant conditions.

### Intracortical source estimation (LORETA)

**Figure 6:**
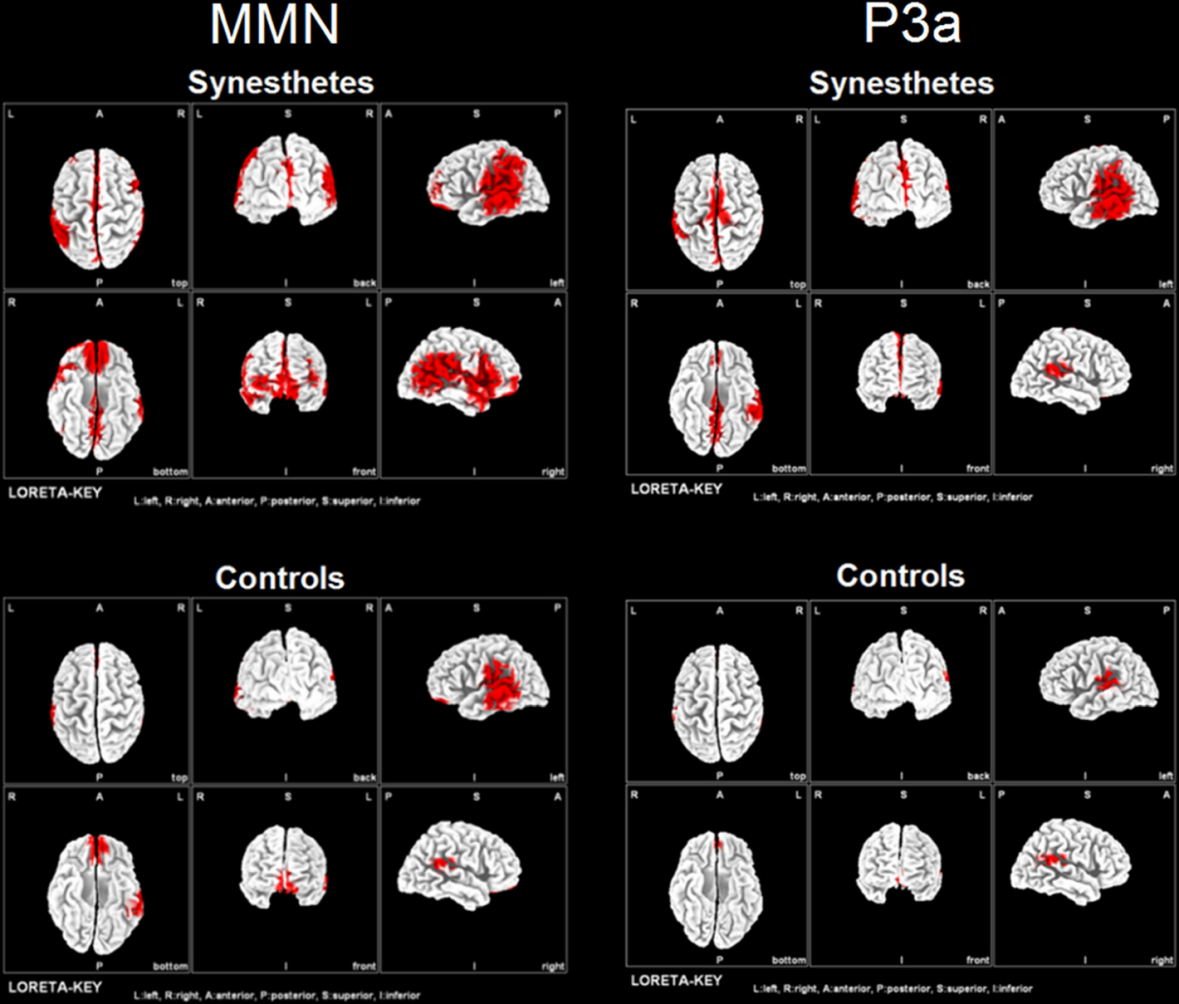
**LORETA source estimation, separately for the MMN and P3a responses to the 264 Hz tone. **An absolute threshold of 0.0004 A/mm^2 ^was used for this analysis.

For the grand average MMN and P3a responses elicited by the 264 Hz tone, the intracerebral sources were estimated separately for synesthetes and non-synesthetes by using LORETA (http://www.uzh.ch/keyinst/loreta.htm; threshold = 0.0004 prop. A/ mm^2^). The MMN elicited in the synesthetes was associated with strong current densities originating from bilateral perisylvian brain regions including the entire auditory cortex, the posterior part of the superior temporal sulcus, the temporoparietal junction, and the inferior and superior parietal lobules. Within the group of non-synesthetes we observed a smaller spatial extension of the perisylvian intracerebral MMN sources with some maxima extending to the left-sided parietal cortex (Figure 6). The same LORETA procedure was applied for estimating putative sources of the P3a response to the 264 Hz tone. In both groups, the strongest current densities for the P3a were located in the bilateral auditory cortex. However, in the synesthetes we also observed areas residing outside the auditory cortex. These areas are the left-sided temporal cortex extending ventrally into the inferior temporal gyrus and mesial brain areas (SMA and precuneus) (Figure 6).

### Voxel-based statistical analyses (LORETA)

**Figure 7:**
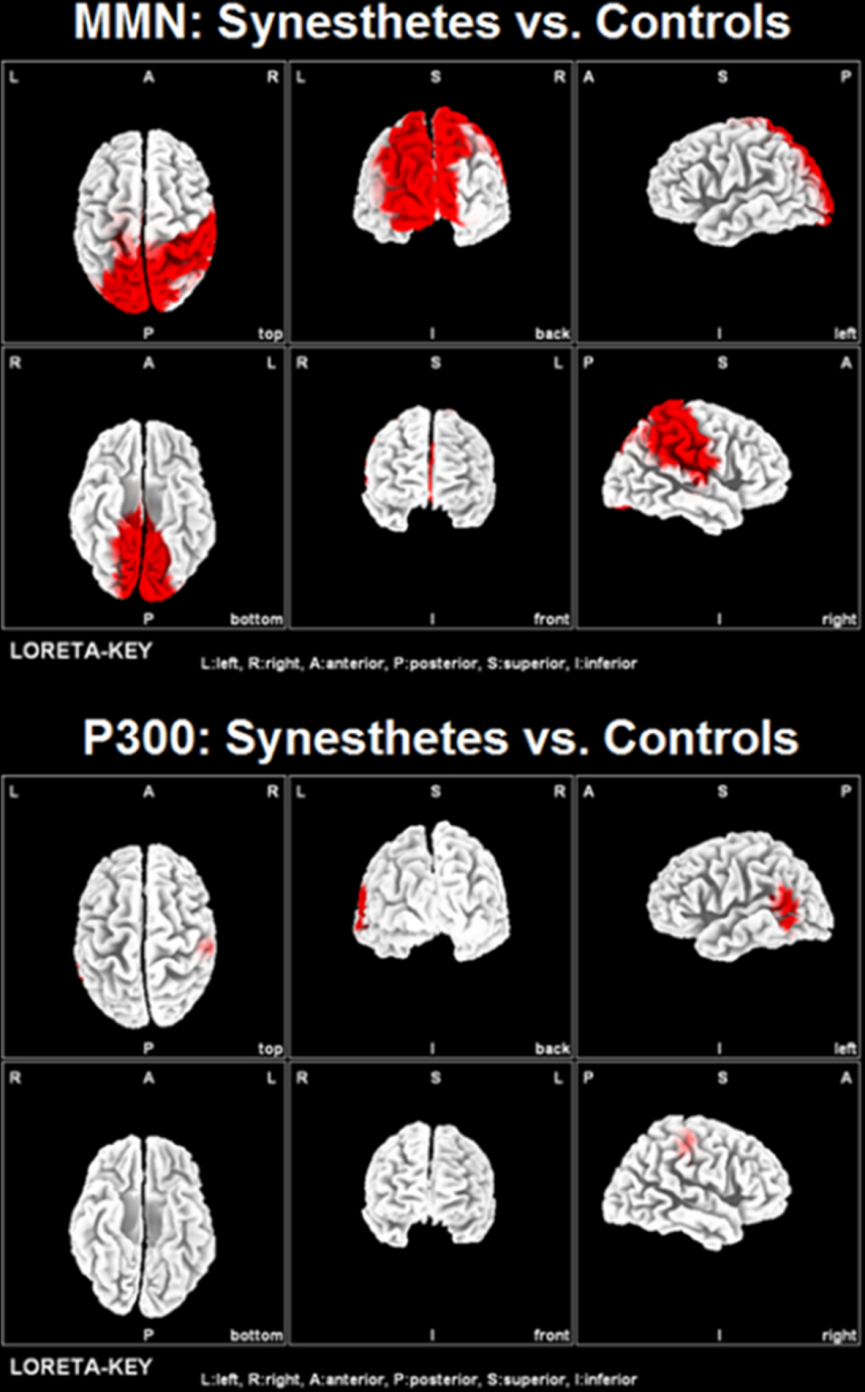
**Voxel-based statistical comparisons between the current densities of the CH synesthetes and non-synesthetes for the MMN and P3a in response to the 264 Hz tone.** A threshold of p < .05 (corrected for multiple comparisons) and a search volume of 1199 voxels was used.

We additionally performed voxel-based comparisons of voxels located in posterior brain parts (excluding the entire frontal cortex and the anterior part of the temporal cortex) between synesthetes and non-synesthetes in response to the 264 Hz tone (MMN and P3a). For the MMN, this statistical analysis revealed significantly stronger current densities in the synesthetes in right-sided auditory-related brain regions, right-sided inferior parietal lobe, bilateral superior parietal lobe, and bilateral in the ventral part of the occipito-temporal cortex (Figure 7, p < .05, corrected for multiple comparisons). For the P3a, between-group comparisons revealed stronger current densities in the synesthetes in left-sided auditory-related regions extending into the angular gyrus (Figure 7, p < .05, corrected for multiple comparisons).

## Discussion

To summarize, in the present work we used the MMN to investigate the timing characteristics as well as the automaticity of tone-colour associations in CH synesthetes. Our hypothesis was that CH synesthetes will elicit increased MMN amplitudes in response to the tones inducing a change in colour perception. In line with our hypothesis, we found larger MMN amplitudes in CH synesthetes, compared to non-synesthetes, in response to the deviants differing at least 1 semitone from the standard (i.e., 416 and 264 Hz tones). These deviants consistently induced different concurrent colours, as provided by the behavioural data. Otherwise, the deviant tone differing from the standard in 1/10 semitone (i.e., 438 Hz) did not elicit larger MMN amplitudes in the CH synesthetes. This is indeed not surprising. In fact, for most synesthetes this tone induced similar colour experiences as those elicited in response to the standard tone.

The differential MMN responses we revealed between the two groups in the time range between 100 and 150 ms after stimulus onset indicate that the concurrent colour perception occurs early in the processing stream. Since the MMN is known to be evoked automatically, pre-attentively, and with no task demands, our findings strongly support the view that tone-colour coupling in CH synesthetes occurs (or starts) automatically and pre-attentively. In both groups, the MMN source estimation (LORETA) revealed strong current densities originating from bilateral perisylvian brain regions, comprising the primary and secondary auditory cortex, the superior temporal sulcus, and the middle temporal gyrus. We also estimated MMN sources originating from the inferior and superior parietal lobules. In addition, voxel-based statistical comparisons between the two groups revealed stronger current densities in right-sided auditory-related brain regions as well as in the inferior and superior parietal lobule of CH synesthetes. The same statistical analysis also yielded stronger bilateral current densities in CH synesthetes in the extrastriate cortex, precuneus, cuneus, and ventral part of the extrastriate cortex. Thus, 100–150 ms after tone presentation, we identified a distributed network which was significantly differently activated in CH synesthetes compared to non-synesthetes. This network comprises brain areas which have previously been identified to be involved in synesthetic experiences, like the parietal and the extrastriate cortex [11-13,15]. Furthermore, since a major source of the MMN was estimated in the auditory cortex (primary and secondary areas), it is possible that the coupling between auditory and visual experiences is driven by brain activity originating from the auditory-related cortex.

To date, there is only meagre evidence for a specific involvement of the auditory system in CHS [1,2,15,35,36]. Whereas visual inputs into the auditory cortex have been described in humans [15,37-39], non-human primates [40,41], and ferrets [42], a modulation of the auditory cortex may also be mediated by thalamo-cortical interactions [43] and/or feedback loops from multisensory brain areas to the auditory cortex [5], or even by multimodal response properties of neurons situated in the primary auditory cortex and posterior belt areas [44]. The few brain-imaging [3,15,35,45,46] and electrophysiological [1,36,47] studies dedicated to investigate the neural underpinnings of CHS have reported conflicting findings with respect to brain responses in the auditory cortex. In fact, some authors provided evidence for a modulation of auditory-related brain regions in CH synesthetes [1,15,35,36], whereas others could not confirm this finding [3,45-47].

Those studies which have used fMRI techniques to study hemodynamic responses in the auditory cortex of CH synesthetes [3,15,35] are partly problematic for several reasons. First of all, it should be mentioned that the fMRI environment is relatively loud (even when using less loud FLASH sequences). This loudness not only disturbs the subjects, but also causes substantial activation in the auditory cortex, and therefore contaminates the stimuli-related activations [48-51]. In addition, the scanner noise will (at least partly) induce concurrent colour perceptions. Consequently, it results unclear whether the identified hemodynamic responses in the auditory cortex are induced by the stimuli or rather by the noise of the scanner. In contrast, the EEG technique is particular fruitful in that it permits to measure brain responses originating from the auditory cortex which are uncontaminated by noise. In previous EEG studies performed with CH synesthetes, differences in the early N1 component of the auditory evoked potential have been identified [1,36]. The associated intracerebral sources were located in the primary and secondary auditory cortex. Together with our data, these previous studies support the pivotal role of the auditory cortex in generating the concurrent colour perception in CH synesthetes. These results are even in line with a previous study of Hanggi and colleagues [2] who analysed the specific anatomical features of the auditory cortex in the multiple synesthete E.S. who is a tone-colour synesthete. Probabilistic fibre tractography revealed hyperconnectivity especially between the auditory and insular cortices. Thus, we may speculate whether the auditory cortex of CH synesthetes is anatomically and functionally stronger connected with the adjacently located insula, and via the insula with higher-order integration centres. This perspective is further supported by a recent resting state EEG study of our group conducted with CH synesthetes [13]. This specific study identified a strong hub in the auditory cortex of CH synesthetes and emphasize that this area is functionally strongly interconnected with other brain areas [13].

In the present work, we also revealed stronger P3a amplitudes at frontal scalp sites in CH synesthetes compared to non-synesthetes. These larger P3a amplitudes possibly indicate that the bottom-up attention network is stronger activated in CH synesthetes. In fact, a P3a response is thought to indicate the actual orienting of attention to a MMN-eliciting sound change occurring outside the current focus of attention [52]. Several authors have located the source (using MEG, EEG, and intracranial recordings) of the P3a response to deviant tones and novel sounds within the auditory cortex. However, also sources in the frontal cortex, parietal cortex, parahippocampal gyrus, anterior cingulate gyrus, and temporoparietal junction have been reported [53-57]. Statistical voxel-based analyses between the two groups indicated stronger current densities related to the P3a in CH synesthetes within the left-sided posterior superior and middle temporal gyrus, and at the posterior end of the superior temporal sulcus in the vicinity of the temporoparietal junction (in the vicinity of the temporoparietal junction). In addition, we observed a spot of stronger current density in the right-sided inferior parietal cortex in CH synesthetes. These brain regions are known to be engaged in the perception of complex sounds [58], audiovisual processing of speech stimuli, and audiovisual integration in general [38,39,59,60].

In the last decades, a vast amount of work has been dedicated to analyse the spatiotemporal dynamics of crossmodal processing in the brain of non-synesthetes (for an overview see [61]). However, the results arising from these previous studies were shown to be heavily dependent on the experimental paradigms used, the nature of the information being combined, the modalities under investigation, as well as on the analytic strategies adopted by the subjects [61]. Nevertheless, meanwhile an increasing number of reports suggests that sensory brain regions are fundamentally involved in integrating visual and auditory information [62-65]. In addition, recent neuroimaging and electrophysiological work conducted with humans [27,66-69] and animals [40,70] highlighted that the synthesis of auditory and visual information can likewise occur in brain regions thought to be sensory-specific. Currently, only a few EEG studies have investigated the spatiotemporal dynamics associated with the crossmodal processing of elementary AV stimuli, such as disks, flashlights, checkerboards, colours, tones, or noise bursts [27,60,65,68,69,71,72]. These previous studies are important in that they provide evidence for the integration of non-linguistic material in sensory as well as associative brain regions of non-synesthetes. Of particular relevance for our work are two previous EEG studies [27,72], which investigated the crossmodal processing of visually presented disks and pure tones and reported early AV interaction effects between 90 and 140 ms post stimulus onset. In particular, the authors could show that the brain responses elicited by the bimodal stimuli corresponded in latency, polarity, and topography to the N1 component of auditory-evoked ERPs, hence indicating a contribution of auditory-related brain regions to the fusion of elementary visual and auditory information.

Our results replicate the previous findings of the few published neuroimaging [3,15,35] and EEG [1,13,36] studies indicating a modulation of brain responses in the auditory-related cortex of CH synesthetes. Otherwise, in contrast to the two EEG studies of Beeli et al. [1] and Goller and colleagues [36], in the present work we revealed that CHS is associated with increased and not reduced auditory-evoked ERP amplitudes. Although the question whether synesthetic experiences are associated with increased or decreased auditory-related brain responses remains to be fully explored, all EEG studies performed with CH synesthetes have in common a relatively early (between 100 and 200 ms) modulation of auditory-evoked ERPs as a consequence of the crossmodal linkage of auditory and visual information. In contrast to these earlier papers, we adopted a passive MMN paradigm which enables to measure brain responses that are more or less uninfluenced by attention. The two previous EEG studies examining CH synesthetes so far used active auditory discrimination tasks. Thus, it could be that the amount of attentional demands necessary to deal with the inducer may have an influence on the depression or enhancement of brain responses in the auditory-related cortex. In fact, whereas we revealed increased bottom-up driven brain responses in CH synesthetes while passively listening to auditory stimuli, Beeli et al. [1] as well as Goller and co-workers [36] found the reversed pattern; namely decreased auditory-evoked ERP amplitudes during tasks in which the participants overtly attended to the auditory stimuli. Consequently, it is possible that increased brain responses in the auditory-related cortex of synesthetes can be attenuated by the engagement of attention to the inducing stimulus. Furthermore, it is plausible to assume that the physical properties of the stimuli, or even the semantic and overlearned contents of the stimulus material, may have an influence on brain responses in the auditory cortex of CH synesthetes [73-75]. Finally, it is also conceivable that increased or decreased auditory-evoked ERPs may be driven by an additional superimposition of positive- or negative-going deflections, as previously suggested by Goller and colleagues [36].

### Implications for current models of synesthesia

Current models of synesthesia postulate that cross-activation between brain areas processing the inducer and the concurrent perception can either be direct (cross-activation / hyper-binding model) or mediated via some other cortical areas (disinhibition model). As explicitly hypothesized by Brang and colleagues [19], the disinhibition model implies a time lag between neuronal activations in those brain areas processing the inducer and generating the concurrent perception. Thus, the activation in brain areas processing the inducer should precede the activation in brain areas processing the concurrent perception. The cross-activation / hyper-binding model on the other hand would argue for simultaneous activation of these brain areas. However, both models do not make explicit assumptions about the activation in brain areas processing the inducer. Here, we provide evidence that brain areas processing the inducer are activated to a different degree in CH synesthetes compared to non-synesthetes. Furthermore, in our study we employed the MMN, which is known to be evoked automatically, pre-attentively, and with no task demands. Since the MMN amplitudes were larger in CH synesthetes, our findings strongly suggest that tone-colour coupling in CH synesthetes occurs or starts early, automatically, and pre-attentively. Additional voxel-based statistical analyses revealed that the increased MMN amplitudes in CH synesthetes were associated with current densities in the right-sided auditory cortex and inferior parietal lobule, as well as with bilaterally stronger current densities in the superior parietal lobule and the ventral occipital cortex. Some of these areas are involved in processing the inducer (auditory cortex), while other brain areas are known to be involved in multisensory processing (e.g., the parietal and ventral occipital areas). Thus, our data demonstrate that a distributed large-scale network is stronger activated in CH synesthetes at an early and pre-attentive stage of processing. A similar finding for the processing of graphemes in grapheme-colour synesthetes has not been reported so far. Thus, it might be possible that CHS is based on different neural mechanisms than other synesthesia variants. One possibility could be that multisensory integration arises within the auditory cortex of CH synesthetes because inputs from other brain areas (visual or parietal areas) directly influence on-going neural oscillations, so that auditory inputs are amplified or modified (similarly as previously been described for the activity of the auditory cortex in macaques [76]). A further possibility could be that distant cerebral areas are bound together to a functional network by neural synchronisation as has been shown for the perception of visual stimuli [77]. From these perspectives the cross-activation / hyper-binding as well as the disinhibition models are not that different since within such a functional network cross-activation, hyper-binding and disinhibition can be explained on the basis of synchronising mechanisms. However, whether these speculations are indeed valid has to be shown in future experiments using different methods.

### Limitations

The main finding of the present study is that the passive exposition to tones induces increased MMN magnitudes in CH synesthetes compared to non-synesthetes. However, the present work seems to be (at a first glance) somewhat inconsistent with previous findings reporting a decrease of auditory-evoked brain responses in synesthetes compared to non-synesthetes in the context of active listening paradigms. Therefore, further studies adopting both active and passive paradigms within the same sample of subjects would be useful to better understand the contribution of auditory-related brain regions to synesthetic experiences as a function of bottom-up and top-down processes. A further limitation of the present work is that we have included too few synesthetes in order to disentangle the possible influences of inter-individual differences. Finally, we are aware of the fact that estimations of the inverse solution on the basis of 32 scalp electrodes provides only rough estimations of the intracerebral sources.

## Conclusions

We found strong differences in the MMN amplitudes between CH synesthetes and non-synesthetes. This result indicates that in CH synesthetes tones and concurrent colour perceptions are processed early and automatically as compound stimuli. These early different electrophysiological responses in CH synesthetes are accompanied by stronger intracerebral activations in the right-sided auditory-related regions, the inferior and superior parietal lobules, and ventral parts of the occipital lobe. We also revealed stronger P3a amplitudes in the CH synesthetes, this result indicating a more pronounced implicit attention orientation to the deviating tones and their concurrent perceptions. These implicit orienting responses are accompanied by stronger current densities in the auditory-related cortex and the superior temporal sulcus region. Thus, we provide objective electrophysiological evidence indicating that changes in colour experiences in response to tones are accompanied, at least in part, by a modulation of early auditory processing steps in CH synesthetes.

## Methods

### Participants

Eleven CH synesthetes (S, two males and nine females, mean age 30.7 ± 7.5 SD), and eleven control subjects (C, two males and nine females, mean age 29.6 ± 8.1 SD) participated in the present EEG study. All subjects were consistently right-handed with the exception of one subject per group who was ambidextrous, as revealed by the Edinburgh Handedness Inventory [78]. Furthermore, five controls and four CH synesthetes were professional musicians, and two subjects per group reported to be absolute pitch possessors (control subjects, primary musical education: two singers, one violinist, one pianist, and one organist; synesthetes, primary musical education: two pianists, one violoncellist, and one flutist). All CH synesthetes we measured reported to experience colours only in response to auditory non-linguistic stimuli. The study was approved by the cantonal ethics committee (Zurich) and conforms to the Helsinki Declaration. Written informed consent was obtained from all participants and the subjects were paid for their participation. None of the participants reported any history of present or past neurological, psychiatric or audiological disorders, and all subjects had an unremarkable audiological status, as revealed by pure tone audiometry (Home Audiometer software, http://www.esseraudio.com/de/home-audiometer-hoertest.html).

### Behavioural data

#### Musical aptitudes

All subjects performed an auditory test in order to examine their musical aptitudes [79]. This specific test consisted of 30 successive trials in which the subjects had to compare pairs of piano melodies, and to decide whether the melodies were equivalent, rhythmically different, or tonally different. This procedure was applied in order to exclude the influence of different musical aptitudes between the two groups on EEG amplitudes.

#### Absolute pitch (AP) test

In order to attest that the two subjects per group who reported to have AP were effectively AP possessors, we performed an in-house test previously used by our group [33]. During the AP test, participants listened to 108 pure sine wave tones presented in a pseudo-randomized order, and were instructed to write down the tonal label immediately after they heard the accordant tone (i.e., while hearing the 4 sec of brown noise). The presented tones ranged from A3 (tuning: A4 = 440 Hz) to A5. The accuracy was evaluated by counting the total number of correct answers. Semitone errors were counted as incorrect responses in order to increase the discriminatory power. Each tone presented during the AP test had a duration of 1 second and the inter-stimulus-interval (ISI) of 4 sec was filled with brown noise. The whole test unit and its components were created by using Adobe Audition 1.5. (http://tv.adobe.com/de/product/audition/). The AP test was performed by using a HP Laptop and presented via HiFi headphones (Sennheiser, HD 25–1, 70 Ω, Ireland).

#### Cognitive capability

In order to rule out differences in intelligence between the two groups, we adopted the MWT-B test [80]. This verbal procedure permits to estimate the mental ability of the subjects in a short time, and was previously shown to correlate fairly well (r = 0.72) with global IQ in healthy adults [80].

#### Test of genuineness synesthesia

In order to verify that the subjects we measured were indeed CH synesthetes, we performed an established colour-consistency test in which all subjects had to select on a computerized colour palette the colours associated with thirteen randomly presented piano tones, each of them presented three times in the frequency range from 261 to 523 Hz [34]. During this test, the subjects were instructed to navigate per mouse over a colour palette and to choose one of 16.7 million different colours that most closely matched their synesthetic experience for each of the presented tones. Since each selected colour can be represented by a single RGB (red-green-blue) vector with values ranging from 0 to 255, scores reflecting the consistency of tone-colour associations can be calculated. In this way it is possible to compare colour-consistency scores between the two groups. This specific test has previously been shown to be sensitive for distinguishing between synesthetes and non-synesthetes [34].

#### Auditory stimuli

The auditory stimuli were taken from a test battery developed for assessing genuineness of synesthesia [34]. From this test battery, we chose the piano tone A (fundamental frequency f0 = 440 Hz) as the standard stimulus and the piano tone C (f0 = 264 Hz, 9-semitone deviant) as the most prominent deviant tone. The motivation for using an A and a C tone was that the synesthetes reported clear, distinct colour sensations while hearing these two tones. Furthermore, in order to manipulate the magnitude of the deviant tones, we artificially reduced the f0 of the A tone by creating three separate semitone graduations. In particular, we created three additional deviants of 438 Hz (1/10-semitone deviant sounding as a slightly mistuned A), 422 Hz (1/4-semitone deviant sounding like a mistuned G#), and 416 Hz (1-semitone deviant being a G#) by using the Praat software (http://www.fon.hum.uva.nl/praat/). This procedure resulted in one standard tone (i.e., 440 Hz) and four deviants (438 Hz, 422 Hz, 416 Hz, and 264 Hz). Since the 264 Hz and 416 Hz tones belong to a novel tone category in comparison to the standard, we hypothesized that only these two deviants would induce a change in colour experience, and therefore elicit increased MMN amplitudes in the CH synesthetes. In fact, these two deviants represent a sort of double deviation differing from the standard in terms of tone category as well as in synesthetic colours. This double deviation should not arise (or in much smaller size) in response to the 422 Hz and 438 Hz deviants, since these two tones primarily deviate in the auditory dimension. All auditory stimuli lasted 200 ms, were registered as 16-bit stereo files, matched for intensity by normalizing the amplitudes, and were smoothed with a rise- and fall-time of 5 ms in order to avoid an abrupt decay (Adobe Audition 1.5. http://tv.adobe.com/de/product/audition/). During EEG measurements all auditory stimuli were delivered binaurally with a sound pressure level of about 70 dB (Digital Sound Level Meter 329, Voltcraft) by using HiFi headphones (Sennheiser, HD 25–1, 70 Ω, Ireland).

#### Experimental procedure

In the present EEG study, we adopted a passive MMN paradigm [29,30]. The subjects were instructed to watch a black and white film in absence of sound and to focus attention on the film while ignoring the auditory presentation of the stimuli. The experiment consisted of five different runs presented randomly across all participants and the two groups, each run consisting of 420 standard tones and 4 × 70 deviants. In each run, the standard stimulus had an occurrence probability of P = 0.6 and each deviant of P = 0.1. The standard and deviant tones were counterbalanced across the five runs in that each of the five auditory stimuli served as a standard tone as well as a deviant tone. Each run started with fifteen standard tones followed by a pseudo-randomized order of all other stimuli. Additionally, each deviant tone was followed by at least one standard tone, the same deviant was never presented successively, and at least two different stimuli were inserted before presenting again a specific deviant tone.

#### EEG recording and analysis

During EEG measurements, the subjects were placed in a chair at a distance of about 100 cm from a monitor and supported their head on a chin-rest in order to reduce movement artefacts. The EEG (32 channels + 2 zygomatic eye channels, subset of the 10/10 system) was recorded with a sampling rate of 1000 Hz and a band pass filter from 0.1 to 100 Hz by using an EEG-amplifier (Brainproducts, Munich, Germany). We applied sintered silver/silver-chloride electrodes (Ag/AgCl) and used the nose position as online reference. The electrode impedance was reduced to < 10 kΩ by using Electrogel conductant. For all steps of digital EEG raw-data processing, we used the Brain Vision Analyser software (Version 1.04, Brainproducts, Munich, Germany).

The data were high- and low-pass filtered offline at 1–20 Hz, and artefacts were removed by using an independent component analysis (ICA) [81] in association with a semi-automatic raw data inspection. For each stimulus, segments of 500 ms duration were created, including a 100 ms pre-stimulus period. Furthermore, a baseline correction relative to the −100 to 0 ms pre-stimulus time period was applied. The averaged brain responses to the standard tones were subtracted from the brain responses elicited by the identical stimuli presented as deviant tones during the different runs. This procedure resulted in difference waves reflecting the MMN and P3a response. Furthermore, we computed multi-subject grand averages (MMN and P3a) for each group and stimulus type. Based on the voltage distribution over the scalp (see Figure 2A and B), and in order to avoid multiple comparisons between neighbouring electrodes as well as to increase the signal-to-noise ratio, nine frontal electrodes were pooled into one region of interest (ROI: F3, Fz, F4, FC3, FCz, FC4, C3, Cz, and C4) [82].

The time windows used for peak-detection were defined separately for each group and deviant condition according to two consecutive global-field-power minima of the corresponding MMN grand averages. The maximal MMN amplitudes for each deviant-condition and subject were selected by using a semi-automatic peak-detection algorithm, and supplementary confirmed by visual inspection. The finding of a genuine MMN was validated by an inversion of polarity that became manifest at the lateral mastoid electrodes TP9 and TP10. Moreover, in order to verify the presence of a negative deflection, all MMNs were statistically tested against zero by using one-sample *t*-tests. The maximal amplitudes of the MMNs were extracted for each participant and deviant-condition. Data were evaluated with the SPSS software (SPSS 19; http://www.spss.com) by computing 2 × 4 ANOVAs with a two-way grouping factor (group: synesthetes vs. controls) and a 4-way repeated measurement factor (deviant: four deviant conditions as independent variables). All post-hoc *t*-tests were corrected for multiple comparisons by using the Bonferroni-Holm procedure [83].

In addition, the peak amplitudes of the P3a component were semi-automatically measured at the frontal ROI position within the 200–350 ms time frame. The time window for analyses was defined according to the global-field-power minima of the corresponding P3a grand-averages. Similarly as done for the MMN amplitudes, these measures were subjected to a 2 × 4 ANOVA with repeated measurements on one factor. In addition, the P3a amplitudes were all tested against zero.

#### Source estimation and voxel-wise statistical comparisons

In order to examine the intracerebral sources of the MMN and P3a responses, the scalp distributions were subjected to the LORETA software (LORETA, http://www.uzh.ch/keyinst/loreta). In a first step, we analysed the intracerebral sources for the MMN and P3a in response to the 264 Hz tone, separately for each group (threshold of 0.0004 prop. A/mm^2^). For these estimations a transformation matrix with high regularization (1e_ 3 * (first eigenvalue)) was used to increase signal-to-noise ratio [84]. In addition, the LORETA solutions for the MMN and the P3a in response to the 264 Hz tone were voxel-wise statistically compared between both groups by using a randomisation test [85]. Since we were not interested in evaluating the entire frontal cortex, the anterior part of the temporal cortex (every voxel anterior to the Heschl gyrus), the motor cortex, and the sensorimotor cortex, statistical comparisons were performed on half of the voxels only (the total number of voxels for the LORETA solution is 2398; thus we tested for significant differences for only 1199 voxels). Thereby, we applied a threshold of p < .05, corrected for multiple comparisons.

## Endnotes

^a^There are two additional models, which are currently not intensively discussed. The first is the *limbic mediation* hypothesis, first proposed by Richard Cytowic and Frank Wood [86], proposing that synesthesia is mediated by the limbic system and especially the hippocampus, on which multiple sensory signals converge. The second is a hybrid model, the so-called *re-entrant processing model* sharing with the cross-activation model the notion of hyper-connectivity between form and colour processing areas in the fusiform gyrus, and suggests, like the disinhibited feedback model, that synesthetic colours require feedback of neural activity that originates in higher-level areas (e.g., anterior inferior temporal and posterior inferior temporal) to V4 [87].

## Competing interest

The authors declare that they have no competing interests.

## Authors’ contributions

LJ conceived the study, the design, and formulated the hypotheses. SE and LJ drafted this manuscript together. SE coordinated the study and contributed to the study’s hypotheses, design, results analysis, and discussion. LR performed the EEG measurements and evaluated the data together with SE LR also contributed to the study’s hypothesis, design, results analysis, and discussion. MM was involved in drafting and commenting the manuscript. All authors read and approved the final version of this manuscript.

## Supplementary Material

Additional file 1 Table S1.
Synesthetic colours (averaged RGB values) perceived by the CH synesthetes in response to the different tones (A tone, 1/10-semitone deviant, 1/4-semitone deviant, 1-semitone deviant, and 9-semitone deviant). Click here for file
