# Supplementary material for: Pre-attentive modulation of brain responses to tones in coloured-hearing synesthetes
Source: BMC Neurosci. 2012 Dec 14;13:151. doi: 10.1186/1471-2202-13-151 (PMC3547775; doi:10.1186/1471-2202-13-151)
Supplement: Additional file 1 — Table S1. Synesthetic colours (averaged RGB values) perceived by the CH synesthetes in response to the different tones (A tone, 1/10-semitone deviant, 1/4-semitone deviant, 1-semitone deviant, and 9-semitone deviant). [file 1471-2202-13-151-S1.docx]

Table 1

|  |  | Spatial locations |  |  |  | Interferences |  |
| --- | --- | --- | --- | --- | --- | --- | --- |
|  | External | Internal | Gaze centred |  | Posture | Sound source | Unaffected |
| S1 |  | x |  |  |  |  | x |
| S2 |  | x |  |  |  |  | x |
| S3 |  | x |  |  |  |  | x |
| S4 |  |  |  |  |  | x |  |
| S5 | x |  |  |  |  | x |  |
| S6 |  | x |  |  |  |  | x |
| S7 |  | x |  |  | x | x |  |
| S8 | x |  |  |  |  | x |  |
| S9 |  | x |  |  | x | x |  |
| S10 |  | x | x |  |  | x |  |
| S11 |  | x |  |  |  |  | x |

Individual modes of synesthetic color experiences. Most subjects described the tone-induced synesthetic colors occurring internally, while a few experienced them externally. Apart from this polarity, one subject described the synesthetic colors occurring in a gaze-centered manner. For many subjects, the spatial locations varied depending on their posture and/or the sound source. Also many subjects reported their synesthetic colors being unaffected by such interfering aspects. Table 1 pictures the mode of experiencing synesthetic colors of each subject.
